# PDZ*scape*: a comprehensive PDZ-protein database

**DOI:** 10.1186/s12859-018-2156-8

**Published:** 2018-04-25

**Authors:** Jitesh Doshi, Raja Reddy Kuppili, Siddharth Gurdasani, Navneet Venkatakrishnan, Amit Saxena, Kakoli Bose

**Affiliations:** 1 Integrated Biophysics and Structural Biology Lab, Advanced Centre for Treatment, Research and Education in Cancer (ACTREC), Tata Memorial Centre, Kharghar, Navi Mumbai, 410210 India; 2 Homi Bhabha National Institute, Training School Complex, Anushakti Nagar, Mumbai, 400094 India; 3 Centre for Development of Advanced Computing (C-DAC), Pune University Campus, Ganesh Khind, Pune, Maharashtra 411007 India; 4 Present Address: Carnegie Mellon University, 5000 Forbes Ave, Pittsburgh, PA 15213 USA

**Keywords:** PDZ-domain, Protein-protein interactions, PDZ-proteins, Database

## Abstract

PDZ-containing proteins comprise one of the most widely distributed protein families playing major role in localization and membrane receptor clustering. They are hence important regulators of signal transduction in cellular pathways. Although knowledge on these proteins has increased exponentially, the existing database ‘PDZBase’ is limited by presence of only 339 proteins as it dates back to 2004 when very little data was available. Thus, lack of exclusive information on this protein family led us to develop PDZ*scape*. ‘PDZscape’ encompasses the complete available information on **58,648** PDZ-containing proteins with their known and putative binding partners on one platform. It has a user-friendly web interface that can be easily queried with external protein identifiers. With unique integration of prominent databases including NCBI, UniProtKB, Swiss-Prot, Pubmed, PDB, STRING, IntAct, KEGG, Pfam and Protein Mutant Database, it provides detailed information on PDZ interactome apart from the customized BLAST option. Most importantly, this database encompasses the mutations and diseases associated with PDZ containing proteins manually curated by our group, thus making it a comprehensive compilation. It also features tools to query the database using sequence (PDZ-Blast) and to find if protein of interest is a PDZ-binding protein. PDZscape is freely available at http://www.actrec.gov.in:8080/pdzscape.

## Background

**Fig. 1:**
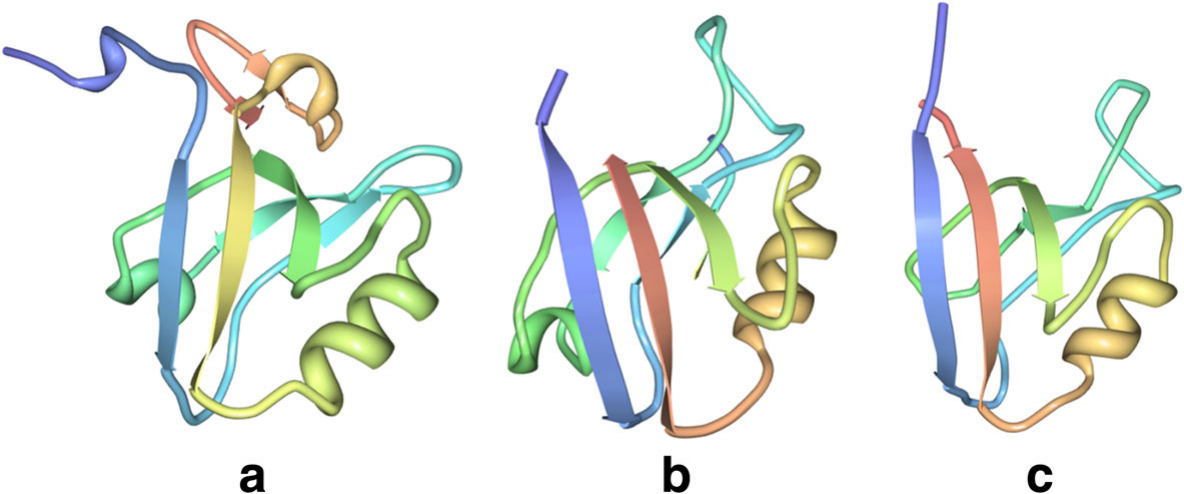
PDZ domains of PSD1, DLG1 and ZO-1 proteins. PDZ domains of (**a**) P*ost synaptic density protein* (PSD95) [PDB ID: 5HDY], (**b**) D*rosophila disc large tumor suppressor* (Dlg1) [PDB ID: 3RL7] and (**c**) Zonula *occludens-1 protein* (zo-1) [PDB ID: 2H3M]. PDZ domains were first discovered in these three proteins

The concept of domains has been one of the core themes in protein studies; one such being PDZ (*P*ost synaptic density protein (PSD95), *D*rosophila disc large tumor suppressor (Dlg1), and *Z*onula occludens-1 protein (zo-1)) - a ubiquitous 70–90 residue protein–protein interaction module. PDZ helps localization and clustering of membrane receptors by binding primarily to their C-terminus in different pathways that drive life [1–4]. Structurally, PDZ domains have a conserved fold, though the secondary structure varies. Over 400 solved structures of PDZ domains suggest that the domain consists of 6 β-strands and two α-helices (one long and one short) (Fig. 1) [3, 5]. The specificity of PDZ-domain-based interactions is determined primarily by the sequence of the C-terminus of the proteins they bind, although few exceptions do exist [6]. Many PDZ containing proteins have more than one PDZ domains, which facilitate multi-protein interactions and thus have been referred to as a ‘Molecular Switch’ [3]. The only existing database ‘PDZBase’ on this domain comprises only about 339 proteins that was last updated way back in 2004 [6, 7] thus creating a need for a database on this domain family that would include the important developments of the last decade. With cellular PDZ proteins as common targets of pathogenic viruses emerging as an important new theme in virology, high throughput and wide spectrum studies on this family of proteins is of significant value [8]. Information about known PDZ proteins, collected together, should be of great importance to research community working with PDZ-proteins and their binding partners. All this information together can help identify role of various PDZ-proteins in different cellular processes.

**Fig. 2:**
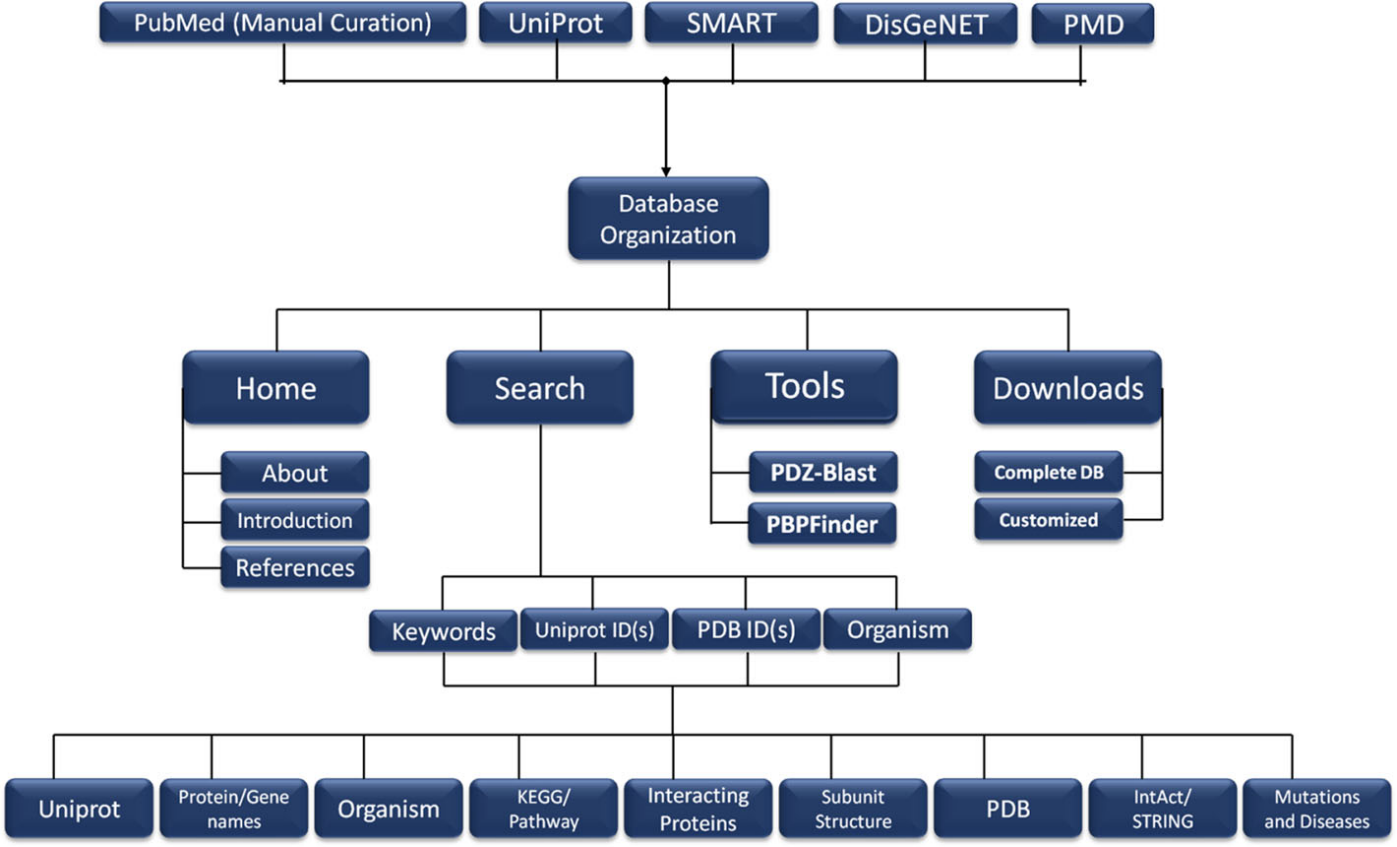
Overview of the PDZ*scape* database. PDZ*scape* is an amalgamation of information from all major databases along with manually curated data on PDZ-containing proteins

**Fig. 3:**
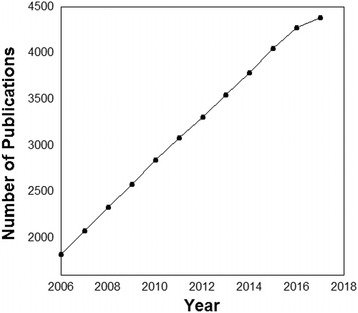
Statistical representation of literature on PDZ proteins. Year-wise analysis shows a sharp increment in research articles published on PDZ-containing proteins over the last decade. (*Source:* Year-wise cumulative number of publications on PDZ from PubMed)

The database **‘PDZ*scape’*** provides information on **58,648** PDZ-containing proteins as well as their known and putative interacting partners on one platform. The user-friendly interface can be easily queried with external identifiers for the protein. Here, unique integration of all prominent databases such as NCBI, UniProtKB, Swiss-Prot, PubMed, Protein Data Bank (PDB), Protein Mutant Database (PMDB), DisGENET, STRING, IntAct, Pfam and KEGG lead to detailed information about PDZ proteins and pathways they are involved in, along with the specific nature of the interaction and the methodology involved. BLAST has been customized for PDZ protein homology search with an additional option of batch downloading the PDZ*scape*. The organization of the database along with its different features is compiled in a self-explanatory flowchart as shown in Fig. 2. It enlists all the well-established and putative interacting partners thus providing a comprehensive picture of this domain family. It also includes information on mutations in the PDZ containing proteins, obtained not only from the PMDB but also through manual curation from literature. Our manually curated data from PMDB and PubMed on the association of PDZ-containing proteins with different pathological conditions significantly enhances the value of the provided information. Additionally, it also provides some tools – PDZ-Blast and in-house developed PBPFinder (PDZ-Binding Protein finder). PDZ-Blast can be used to find similarity between protein of interest and PDZ-proteins from PDZscape. PBPFinder helps to find whether a protein of interest is a known PDZ-binding partner. It also provides an option to scan small peptides for presence of PDZ-binding motif. Lastly, with the amount of data generated in the last decade (Fig. 3), and its involvement in a myriad of cellular pathways, it is essential to have updated information exclusively on this very important class of protein-protein interaction domains. Most importantly, this data will help in delineating various cellular functions and pathways involving PDZ-proteins. PDZScape can also be used as a tool to understand pathophysiology of diseases associated with aberrations in PDZ-proteins.

## Construction and content

### Software tools

MySQL Administrator Tool (Workbench 6.0 CE), Eclipse, Apache Tomcat 9 server, Java 1.8.

### Data sources

The PDZ protein data and related information such as sequence, organism information, available structures, localization and related literature were obtained from publicly available Uniprot-KB/Swissprot (release 2017_20) database. Uniprot-KB/Swisprot being the largest and up-to-date manually curated database of proteins was the most important data source for PDZ*scape*. The entire PDZ domain containing data was manually downloaded from Uniprot-KB as flat files and the other related information was extracted out in a CSV (Comma Separated Values) file using Perl regular expressions. This CSV file was further used for the creation of tables in a database using MySQL server. Various information on PDZ*scape* proteins such as their Gene information-Gene IDs, structural information-PDB IDs [9], protein-protein interaction information- STRING IDs [10] and Pathway information - KEGG IDs were taken from their respective databases. The number and organizations of PDZ domains have also been included separately. Information on PDZ-interacting proteins was obtained from IntAct database [11] that is the largest molecular interaction network database, which includes all the interacting partners of a protein. Sequences of all PDZ-containing proteins were assimilated to form the source database of PDZ-BLAST (Basic Local alignment Search Tool). The stand-alone BLAST (2.6.0+) [12, 13] feature was downloaded from NCBI website and integrated in the database. For searching and homology scoring functions, default matrices of BLAST [12, 13] were used. Known mutations were extracted from PMDB and some of them were manually curated from literature. The phenotypic implications associated with each of these mutations, if any, have also been included in the PDZ*scape* output. Extensive manual curation was also performed for obtaining information on the association of PDZ containing proteins with different pathological conditions. Currently, more than 300 entries have been curated with the known mutations and their association with various diseases. This information is available for Human PDZ proteins and will be periodically updated. Manual curation was performed using literature search on PubMed and filtering the papers based on proven disease associations from experimental studies. Literature reports for proteins, where only mutations have been reported without any information on associated diseases, have also been included in the database wherever relevant. It has been observed that disease-association in PDZ-containing proteins is not limited to mutations but depends on other factors as well that include over-expression, chromosomal deletion, inhibition etc. These diseases have also been reported in this database. The entire data takes into consideration all PDZ proteins and can be retrieved in ‘*.csv’* format from download page.

### PBPFinder

To facilitate reverse search and find whether the protein of interest is a PDZ-interacting partner, a simple tool, based on sequence similarity and ID mapping has been developed in Java, which takes UniProt ID of a protein as an input and finds whether it is a PDZ-binding protein. In both protein and peptide mode, this tool scans the given sequence for presence of known PDZ-binding motif, which are stored as regular expressions, based on reports from the literature [14]. PBPFinder is a simple tool that first scans the database of known PDZ-binding proteins with given sequence or Uniprot ID and database of known PDZ-binding motifs in order to report the possibility of given protein being a known or putative PDZ-binding protein.

### Database integration

PDZ*scape* database was developed with JavaScript using Eclipse Juno software development environment. For data integration and parsing, programs were written in JavaScript and Perl. These programs were used to search and parse the data on PDZ proteins and their interacting partners from flat files to create output files in MySQL Tables. All MySQL queries to the databases were implemented in a Javascript page using Java-based data access technology (JDBC) connection and have been uploaded on Linux-based TomCat 9.0 Apache server. The linking of entire data integration server was done using Eclipse Software Development Environment (SDE). Eclipse is a multi-language Integrated Development Environment (IDE) comprising a base workspace and an extensible plug-in system for customizing the environment, which is widely used for database and software development. This database has been constructed using various sources of PDZ-containing protein sequences, and information on their structure and interacting partners. The information so compiled not only includes well established interacting partners but also putative ones that can prove to be useful leads for further investigation by researchers. This wide range of data was amalgamated together to form a new and comprehensive knowledge-base for PDZ proteins.

## Utility and discussion

**Fig. 4:**
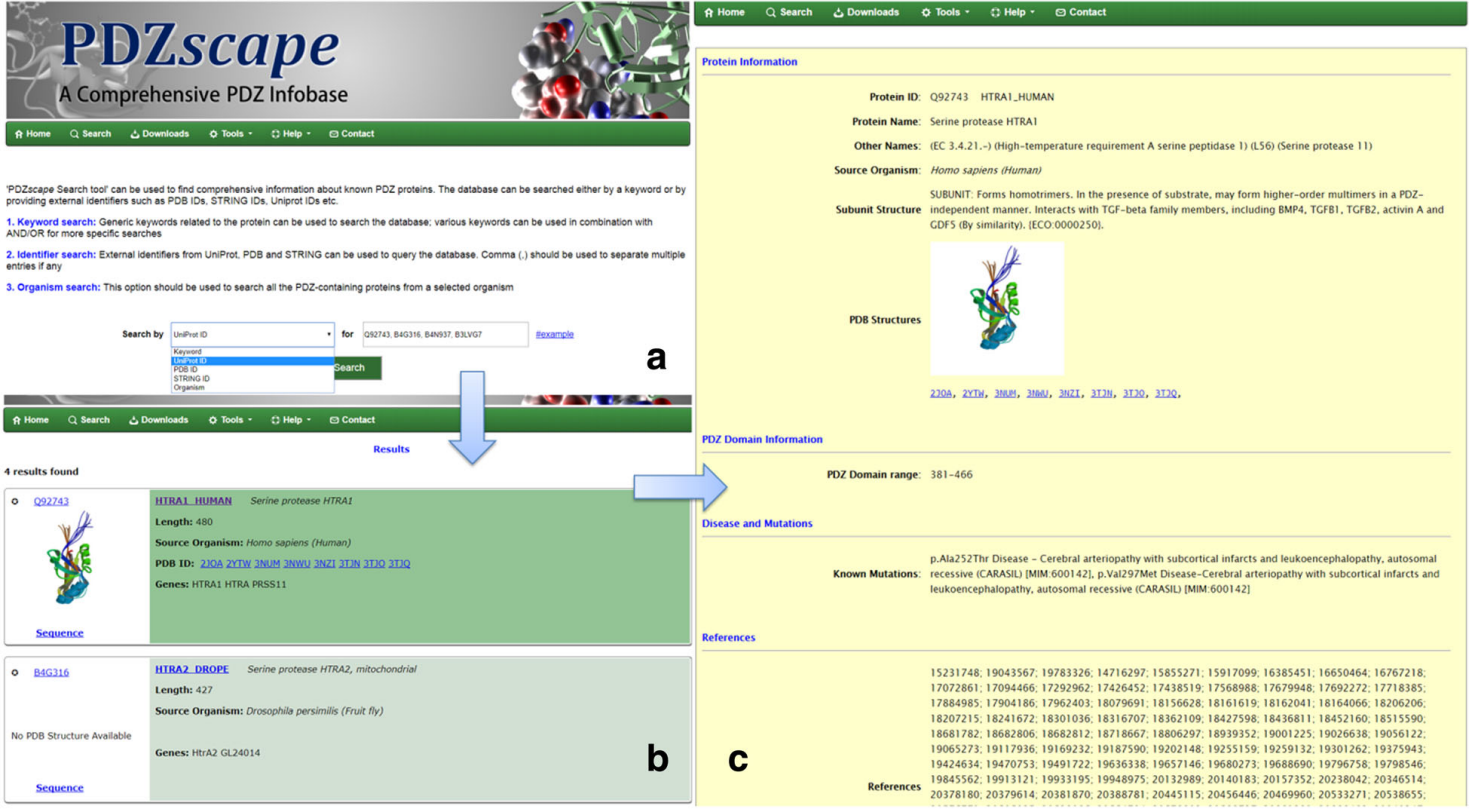
The PDZ*scape* graphical user-interface (GUI) and output: The screenshots represent the output generated by the database search using query protein. (**a**) Search page of PDZscape allows searching by Keywords, Uniprot IDs, and PDB IDs or organism names. (**b**) Summary of proteins related to search criteria provided. (**c**) Individual protein entry page with available information

PDZ*scape* Database is available online through ACTREC Home Page (http://www.actrec.gov.in/) under ‘Database & Tools’ (http://www.actrec.gov.in:8080/pdzscape) and has an easy-to-browse web interface. End user can retrieve complete information of a particular protein entry using any of the following: Keywords, Uniprot IDs, String IDs, PDB IDs, organism names or protein sequence. Search tool provides easy access to the database using various criteria as shown in Fig. 4. Keywords can be used to search the database using generic terms such as protein names, gene names or other related information for more specific search. Keywords can also be used in combination with ‘AND/OR’ for filtered results. Other search methods provide access to database using one or more external identifiers like Uniprot ID, PDB ID etc. Multiple IDs can also be entered separated by commas. Quicker access to the data is also possible where user can reach to a particular protein entry in PDZ*scape* using URL: http://www.actrec.gov.in:8080/pdzscape/protein.jsp?accID=*UniProtID*, where term *UniProtID* should be replaced by protein identifier of required PDZ-protein. Figure 4 shows sample query string and the output of the search in PDZScape. Our manually curated data from PMDB and PubMed on the association of these proteins with different pathological conditions significantly enhances the value of the provided information. PDZ-BLAST facility allows user to search PDZ*scape* using sequence of the protein of interest, which is accessible to the end-user through the hyperlink provided in the main menu. The query sequence can thereby be entered either manually by pasting FASTA sequence in the respective search box or by uploading a FASTA sequence file. Results can be downloaded in the pdf format from the BLAST result page. ‘*Download*’ option can be used to retrieve the complete database in CSV format; customized subsets can also be downloaded from this link. The links provided in the database highly expand the annotations of the PDZ*scape*, and will enable end-user to have access to every bit of required information on a single arena. PDZ*scape* is designed to include periodic updates and inclusion of new features to make it a useful portal for biologists. PBPFinder currently works on regular expressions for motifs described in previous literature. Development of robust machine learning model for prediction of PDZ-binding proteins is in process and will be a valuable addition to this database. While PDZbase is a well-known portal for PDZ information, it contains a limited number of PDZ-proteins with focus on the details of protein-protein interactions that perhaps needs updating. On the other hand, PDZscape encompasses all PDZ-proteins with known and putative binding partners.

## Conclusion

**Fig. 5:**
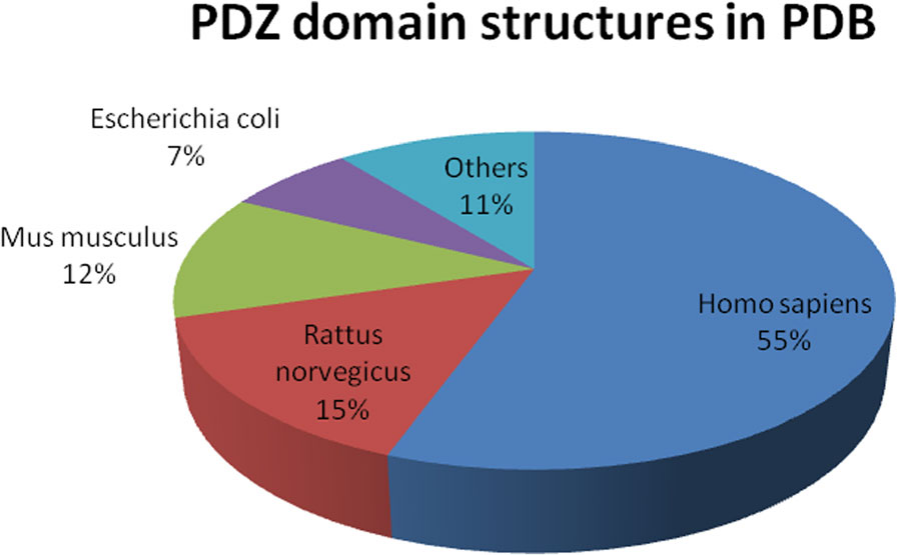
Pie-chart representation of the structures solved for PDZ proteins. A total of 551 structures for the PDZ domains have been deposited till date with the distribution in different organisms (human PDZ proteins are pre-dominant). (*Source:* Number of PDZ domain structures deposited in Protein Data Bank (PDB))

A large number of genes have been annotated over the last decade with the exponential increase in the number of depositions on PDZ proteins in NCBI and other sources. This provides an exciting as well as challenging prospect of studying these newly identified and annotated proteins under a single platform. The plethora of research endeavors that have undertaken in the last decade on this protein domain underscores its importance in critical cellular processes and association with several disease conditions. Categorizing proteins, though important is a relatively simple job compared to their structural and functional characterization. With large number of PDZ-proteins being identified and structurally characterized (Fig. 5), there was a dire need of a compiled resource on these proteins that include information on their involvement in vital cellular processes, and their disease association.

The existing PDZ domain database, PDZBase currently contains approximately 339 proteins and their interactions [6]. The information on interacting partners in PDZBase is based on in vivo or in vitro experiments. However interactions obtained from high throughput methods alone were excluded [6]. On the other hand, PDZScape comprises experimental as well as putative/predicted interacting partners of PDZ proteins. PDZ-ligand interaction network available from this database would help link PDZ-proteins with cellular pathways and associated diseases thus providing possible leads for therapeutic interventions and target identification. In the current context, where voluminous data is being generated increasingly, this database provides minimal information on PDZ proteins that leads from predicted studies. Therefore, information available through manual curation for known mutations and disease associations would provide a valuable resource on PDZ-proteins.

## Availability and requirements

Database is freely available for public use at http://www.actrec.gov.in:8080/pdzscape/.

## Abbreviations

BLAST: Basic Local Alignment and Search Tool
IDE: Integrated Development Environment
PBPFinder: PDZ-Binding Protein finder
PDB: Protein Data Bank
PDZ: *P*ost synaptic density protein (PSD95), *D*rosophila disc large tumor suppressor (Dlg1), and *Z*onula occludens-1 protein (zo-1)
PMDB: Protein Mutant Database
SDE: Software Development Environment

## References

[1] Boeckmann B, Bairoch A, Apweiler R, Blatter MC, Estreicher A, Gasteiger E, Martin MJ, Michoud K, O'Donovan C, Phan I (2003). The SWISS-PROT protein knowledgebase and its supplement TrEMBL in 2003. Nucleic Acids Res.

[2] Campagne F, Bettler E, Vriend G, Weinstein H (2003). Batch mode generation of residue-based diagrams of proteins. Bioinformatics.

[3] Dueber JE, Yeh BJ, Chak K, Lim WA (2003). Reprogramming control of an allosteric signaling switch through modular recombination. Science.

[4] Fan JS, Zhang M (2002). Signaling complex organization by PDZ domain proteins. Neurosignals.

[5] Lee H-J, Zheng JJ (2010). PDZ domains and their binding partners: structure, specificity, and modification. Cell Commun Signal.

[6] Beuming T, Skrabanek L, Niv MY, Mukherjee P, Weinstein H (2005). PDZBase: a protein─protein interaction database for PDZ-domains. Bioinformatics.

[7] Hung AY, Sheng M (2002). PDZ domains: structural modules for protein complex assembly. J Biol Chem.

[8] Javier RT, Rice AP (2011). Emerging theme: cellular PDZ proteins as common targets of pathogenic viruses. J Virol.

[9] Berman HM, Westbrook J, Feng Z, Gilliland G, Bhat TN, Weissig H, Shindyalov IN, Bourne PE (2000). The Protein Data Bank. Nucleic Acids Res.

[10] Szklarczyk D, Franceschini A, Kuhn M, Simonovic M, Roth A, Minguez P, Doerks T, Stark M, Muller J, Bork P (2011). The STRING database in 2011: functional interaction networks of proteins, globally integrated and scored. Nucleic Acids Res.

[11] Orchard S, Ammari M, Aranda B, Breuza L, Briganti L, Broackes-Carter F, Campbell NH, Chavali G, Chen C, del-Toro N (2014). The MIntAct project--IntAct as a common curation platform for 11 molecular interaction databases. Nucleic Acids Res.

[12] Altschul SF, Gish W, Miller W, Myers EW, Lipman DJ (1990). Basic local alignment search tool. J Mol Biol.

[13] Camacho C, Coulouris G, Avagyan V, Ma N, Papadopoulos J, Bealer K, Madden TL (2009). BLAST+: architecture and applications. BMC Bioinformatics.

[14] Tonikian R, Zhang Y, Sazinsky SL, Currell B, Yeh J-H, Reva B, Held HA, Appleton BA, Evangelista M, Wu Y (2008). A specificity map for the PDZ domain family. PLoS Biol.

